# Reproductive Seasonality of the Antarctic Sea Pen *Malacobelemnon daytoni* (Octocorallia, Pennatulacea, Kophobelemnidae)

**DOI:** 10.1371/journal.pone.0163152

**Published:** 2016-10-12

**Authors:** Natalia Servetto, Ricardo Sahade

**Affiliations:** Marine Ecology Laboratory, Instituto de Diversidad y Ecología Animal [Consejo Nacional de Investigaciones Científicas y Técnicas–Universidad Nacional de Córdoba (CONICET-UNC)], Facultad de Ciencias Exactas, Físicas y Naturales, UNC, Avenida Vélez Sarsfield 299, 5000, Córdoba, Argentina; Laboratoire Arago, FRANCE

## Abstract

The pennatulid *Malacobelemnon daytoni* is one of the dominant species in Potter Cove, Antarctica. Its abundance and range of distribution have increased in recent years probably related to climate change mediated alterations of environmental factors. This work is the second part of a study dealing on the reproductive ecology of *Malacobelemnon daytoni*, and aims to assess its reproductive seasonality over a two-year period. Sampling was carried out every month during 2009–2010 and samples were examined by histological analysis. Gametogenesis exhibited a seasonal pattern evidenced by the maturity stage index (MSI) and the number of mature oocytes and cysts throughout the year. Immature oocytes and spermatocytes were present year-round, but maturation was seasonal and it seems that more than one spawning per year was possible. These spawnings could be more linked with suspended particulate matter (SPM) (probably available via resuspension events) than with primary production pulses. This idea reinforces the hypothesis that winter time is not so stressful, in energy terms, in Potter Cove, which seems to depend on energy sources other than local phytoplankton production. There was not a strong inter-annual variability between the reproductive characteristics analyzed in 2009 and 2010; the only variable different was the size of oocytes (higher in 2009), suggesting different energy availability in each year, related with a higher concentration of SPM in 2009 (although it was not significant). *Malacobelemnon daytoni* could be the first reported Antarctic suspension feeder species that presents a reproductive cycle with more than a spawning event per year. This strategy would help to explain the success of this species in the Potter Cove ecosystem and in high ice-impacted areas.

## Introduction

Antarctic marine benthic communities are subject to several specific environmental variables, such as low but stable temperatures and a continuous dark or light regime for some months driving the probably most important factor in influencing the biology and ecology of shallow marine organisms, the marked seasonal pattern of primary production[1,2]. The influence of this seasonality may vary with the position in the food web, with herbivores being more affected, while groups in higher levels of the food web such as scavengers or carnivores may be less correlated to seasonal pulses[1,3,4]. Reproductive seasonality of Antarctic benthic invertebrates generally respond to this idea. In high latitudes many comparable taxa show a trend towards brooding; there is a tendency to species with lecithotrophic larvae; longer gametogenesis and larval development, compared with their temperate and tropical counter parts[5–8].

Suspension feeders are among the most abundant and dominant groups in the Antarctic benthos, however reproduction studies dealing with these taxa are still scarce, especially in octocorals, which are important members in benthic communities from shallow coastal to deep waters around Antarctica[9–11]. Soft corals are predominantly gonochoric (more than 80%; contrasting with mostly hermaphroditic species in scleractinian), and mode of reproduction is divided between broadcast spawning and the 2 forms of brooding: internal and external [12]. Octocorals also show a correlation among reproductive modes and climate. The spawning strategies and continuous reproductive cycles are very common in tropical waters (with the exceptions of reefs species that can show marked spawning seasonality in typical multi-species synchrony events), while brooding and seasonal strategies are more common in colder waters [12,13].

Pennatulaceans are a morphologically diverse group with an estimated 200 or more valid species and are important members of the sessile megafauna of soft bottom habitat. The geographic and bathymetric distribution is very wide, from polar seas to the tropics, and from intertidal flats to over 6100 m in depth [14]. In contrast to other octocorals, up to date only broadcast spawning and lecithotrophic larvae species were reported [15–26]. On the basis of the relationships of these traits with climate, these characteristics were recently confirmed in *Malacobelemnon daytoni* [27]. With this, including an Antarctic species, the widest latitudinal gradient in reproduction studies among pennatulacea was covered. It permitted to evaluated the Thorson´s rule since a high latitude species is a good candidate to test if internal fertilization would have been possible in the group[28]. In this way, Servetto et al. [27] supported the idea of broadcast spawning could be a fixed trait in the group. Among pennatulid species, from one single spawning per year to long, continuous breeding activity have been observed [15–26].

The pennatulid *Malacobelemnon daytoni* [29]is one of the most abundant specie in a shallow benthic community in Potter Cove (South Shetland Island) Antarctica. Its abundance and the distribution range of this specie has significantly increased in the last years. It is striking because it was observed in areas with the higher sediment influence and areas highly affected by the impact of the ice [28,29]. While burying behavior into the sediment was observed in some pennatulids, this has not been observed by divers in *Malacobelemnon daytoni* to date, so, this species must have another strategic to cope with the impact of ice. Consequently, the main aim of this work was to investigate the reproductive seasonality of *Malacobelemnon daytoni*, a species that makes an important contribution to the structure of Potter Cove benthic communities. This may support or disprove the hypothesis that this species present a high population turnover to cope with the impact of ice. And in this way assess whether the reproductive strategy of this species could help to explain its ecological success in the Potter Cove ecosystem.

## Materials and Methods

### Study site and sampling

This work was carried at Potter Cove (62°14’ S, 58° 38’W),using the facilities of Dallmann laboratory in the Argentinean Carlini station. Potter Cove is an inlet close to the entrance of Maxwell Bay, one of the 2 main fjords at Isla 25 de Mayo (King George Island) (see Servetto et al. [27]).

Samples (284 colonies between females and males) were collected monthly from 15 to 22 m between January 2009 and December 2010by SCUBA diving and were preserved in 4% formalin sea water solution [27].

All the available colonies fixed were rinsed with fresh water; the rods were decalcified in diluted HNO_3_, and were then embedded in paraffin. The material was serially sectioned (3–4 microns) and stained with Hematoxilin-Eosin for histological examination. Longitudinal histological cuts of the complete colonies were examined under a stereomicroscope (Labomed CZM4) provided with a digital camera(Nikon COOLPIX E995) and analysed using the imaging software ImageJ (1.48v). Some samples were not analyzed as not all histological sections were in a good condition, the number of colonies included in this analysis are explicit in Table 1.

**Table 1:** no. of male and female colonies studied by month in 2009 and 2010. no. cysts and oocytes examined by colony.

Month	Number female colonies examined	Number oocytes measured	Number male colonies examined	Number cysts examined	Colonies without reproductive cells
**2009**					
January	6	440	3	78	
February	5	302	6	440	4
March	10	712	9	850	
April	4	146	2	192	**1**
May	5	233	5	781	
June	4	308	4	117	**1**
July	5	209	4	169	
August	5	334	nd.	nd.	
September	3	93	6	101	
October	1	nd.	2	199	**2**
November	5	211	3	169	**3**
December	11	442	6	424	
Total	64	3430	50	3520	**9**
**2010**					
January	5	135	4	259	
February	2	59	7	572	
April	2	168	3	427	
May	7	417	3	413	
July	6	280	4	114	
August	4	265	3	125	
November	4	103	1	67	
December	6	469	2	108	
**Total**	**36**	**1896**	**27**	**2085**	

***nd*.:** no data

The female colonies at the present work are the same that in Servetto el at. [27].

### Histological analysis

The length of all the colonies was measured from the base of the peduncle to the tip of the rachis. Each colony was divided into three zones as in Servetto et al. [27]: base, middle and apical (adapted from[15,16,21]). All oocytes and cysts of the three longitudinal sections were counted and oocyte size (Feret diameter; [30]) was measured only in those oocytes sectioned at the nucleolus level [21,31,32].

Gametogenesis was classified both by histological characteristics and by size of the oocytes using the same terminology that in Servetto et al. [27] (stage I, early growth oocytes <70 μm; stage II: 70 to 130 μm; and stage III, mature oocytes >130 μm). The size of the cysts was measured and the large cysts were considered most mature [21]. Spermatogenic stages were defined as follows: early growth (Stage I); small spermatocysts, empty with low density of spermatozoa (< 150 μm). Growth (Stage II) (150 to 300 μm): increased density of spermatozoa; and mature (stage III) spermatocysts > 300 μm, lumen filled with spermatozoa(S1 Fig).

### Fecundity and Maturity stage index

Fecundity is calculated in order to make inter-annual comparisons (2009–2010) in a standardized manner. The effective relative fecundity (ERF) was calculated using the terminology adapted from Baillon et al. [15]and Mercier and Hamel [33] as:number of mature cysts and oocytes/colonies, expressed as number oocytes or cysts.colony^-1^. The maturity stage index (MSI) represents a quantitative measure of gametogenic maturity on a continuous scale that lends itself to statistical analysis[15,34]. It was determined for male and female colonies, using the equation adapted from Baillon et al. [15]: ln[(ERF × colony length^−1^) × total oocyte or spermatocyst volume × 0.01 + 1]. Total oocyte or spermatocyst volume corresponded to the sum of individual oocyte or spermatocyst volumes in the colony; the volume was determined as: π × diameter^3^/6.

The ERF, MSI and the average size of the oocytes and cysts were compared between two years studied (2009–2010) in comparison with several environmental variables studied by Schloss et al. [35] in those years.

In the framework of the Research project PICTO Antártida Ref 36326. ANPCyT-DNA (‘‘Antarctic benthic communities: an interdisciplinary approach to analyze the possible impact of global warming"), the Environmental and Tourism Antarctic Management Program of the National Direction of the Antarctic (DNA) in the Republic of Argentina, has issued the appropriate permissions to all the stages of this research:

- Taking and harmful interference and introduction of species (under art. 3, Annex II of the Madrid Protocol, Law 24216)
- To the Specially Protected Area Nu132 ‘‘Peninsula Potter” (under art. 7, Annex V of the Madrid Protocol, Law 25260).

Both of these permissions properly followed the regulations in force.

### Statistical analysis

Normality was tested using Shapiro-Wilk test, and homogeneity of variances was examined using Levene's Test. When variances were not homogeneous after transformations, non-parametric Mann-Whitney and Kruskal-Wallis tests were used and then multiple range tests as described by Conover [36]were used *post hoc*.

Statistical analyses were carried out using Infostat 2012.

## Results

### Seasonality and spawning

Reproductive cells were not found in all sampled colonies. There were four colonies in February, three in November 2009, two in October and one in April and June, with no oocytes or cysts (Table 1).

**Fig 1:**
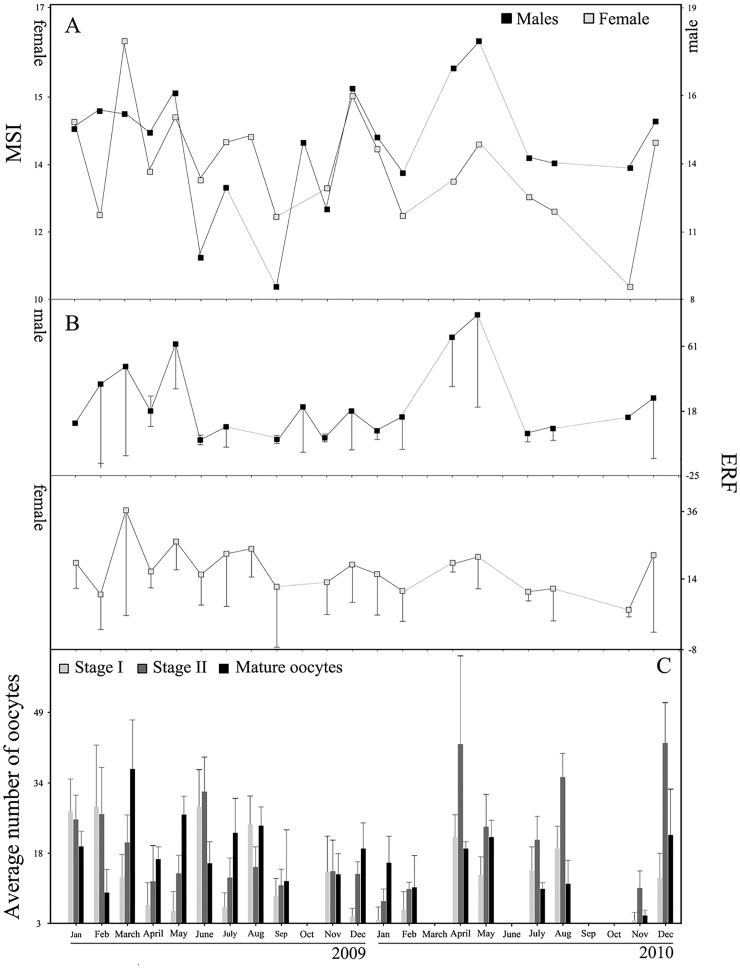
Maturity stage index and fecundity. (A) Maturity stage index (MSI); (B) effective relative fecundity (ERF) in females and males and (C) average number of mature, at stage I and II oocytes, between January and December 2009 and 2010. Vertical bars indicate ± SE.

All female colonies with reproductive cells presented oocytes in the three stages, I, II and mature oocytes were found. The lower female indexes (MSI) were registered in February and September 2009 (MSI = 12.31 and 12.27 respectively) and February and November 2010 (12.29 and 10.61 respectively) (Fig 1A). A similar pattern was observed in males with the lower MSI observed in September 2009 (MSI = 9.31), MSI was also low in June and November 2009 (MSI = 10.45 and 12.35 respectively). In 2010 MSI showed higher values than 2009 and decayed in February (MSI = 13.75) and less markedly in July (14.36) (Fig 1A).

**Fig 2:**
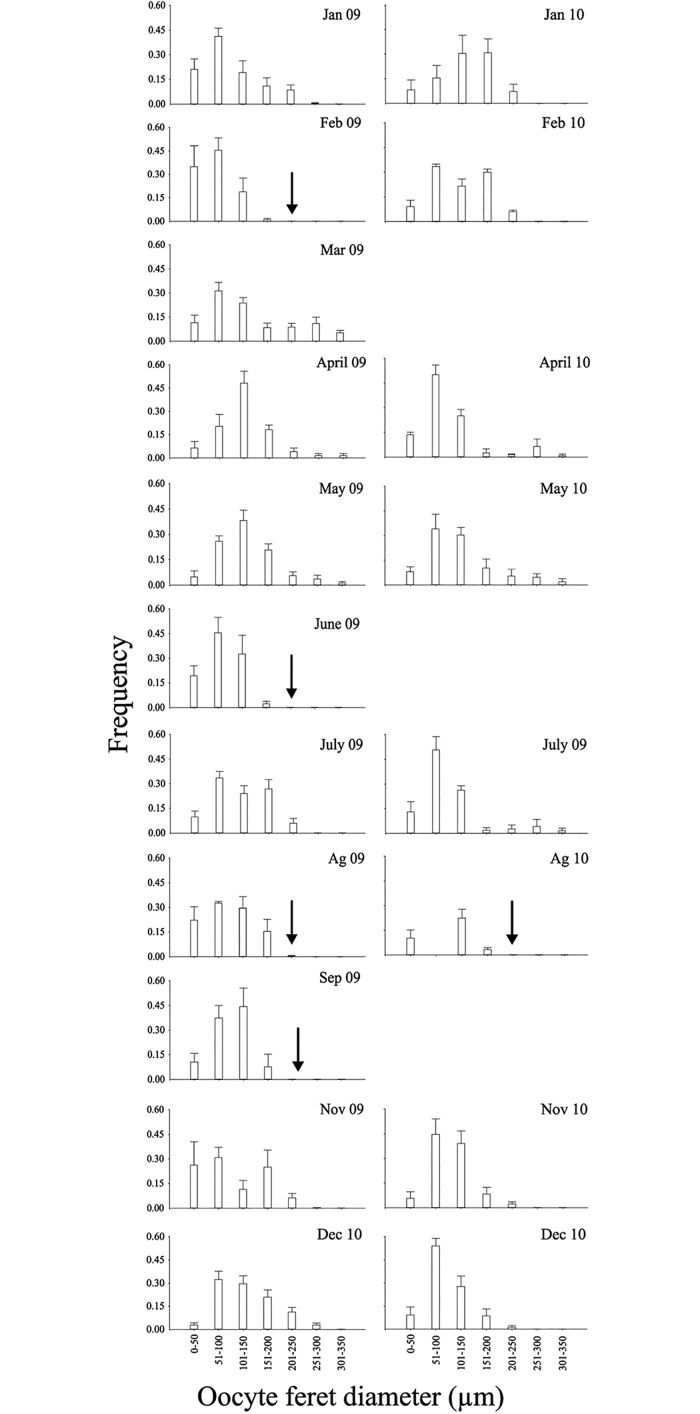
Relative frequency of oocyte size classes in colonies collected between January 2009 and December 2010. The *vertical arrow* indicates the absence of mature oocytes >200 μm. Vertical bars indicate ± SE.

The proportions of stage I, II and III oocytes varied over the sampling period. The highest number of female ERF was registered in March 2009 (mean = 37.10; SD = 34.15) and the lowest in February and September 2009 (mean = 9.80; SD = 11.34; mean = 12.33; SD = 19.66 respectively). In 2010 the lowest value was registered in February and November (mean = 11; SD = 9.90; mean = 4.75; SD = 2.22 respectively) (Fig 1B). Similarly in November 2010 the lowest number of oocytes in Stage I (mean = 3.75; SD = 3.30) was observed, while the highest was found in February 2009 (mean = 28.8; SD = 30.23) (Fig 2C). Small oocytes (stages I and II) and mature oocytes were observed along all the studied period, and mature oocytes bigger than 200 μm, were registered in February, June, August, September 2009 or August 2010 (Fig 2).

**Fig 3:**
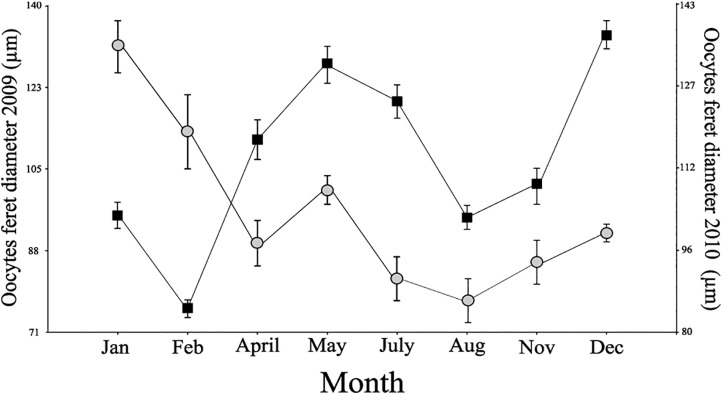
Oocytes feret diameter. Mean oocyte feret diameter (μm) in 2009 (black box) and mean oocyte feret diameter in 2010 (gray circle). Vertical bars indicate ± SE.

Table 2 compares several variables of the two studied years, 2009 and 2010. The average size of oocyte feret diameter was the only variable with significant differences between the sampled years, being slightly higher in 2009 than in 2010(Fig 3 and Table 2). Most of the variables analyzed were also higher in 2009 than in 2010 although not statistically significantly (female ERF and MSI and spermatic cysts size).

**Table 2:** Comparison of several environmental variables and reproductive characteristics between 2009 and 2010. Mann Whitney non-parametric analysis was used and *p*< 0.05 indicated a significant difference.

	Mean (2009)	Mean (2010)	*W*	*p-value*
Oocytes feret diameter	113.15 (± 1. 07)	100.48 (±1.38)	4772010.50	0.0001 **(*S*)**
ERF (f)	21.70 (± 18. 12)	15.61 (± 13.37)	1543	0. 0614
MSI (f)	13.90 (± 1. 26)	12.88 (± 1.15)	59	0.0908
Spermatic cysts size (μm)	272.36 (± 116. 2)	270 (± 105.00)	5763368	0.6218
ERF (m)	24.66 (± 38. 12)	25.48 (± 35.7)	1131.5	0.3978
MSI (m)	14.32 (± 2. 63)	15.50 (± 1.93)	86	0.6203
Chlorophyll-a (mg-m^-3^)	0.48 (± 0. 47)	0.89 (± 1.55)	5569	0.5417
SPM (mg.m^-3^)	7.46 (± 8. 13)	6.25 (± 5.57)	2260	0.5574

***ERF*:** effective relative fecundity*;* ***W*:** test statistic*;* ***S*:** significant; ± *SD*.***MSI*:** Maturity stage index; **f:** female; **m:** male. Data of the Chlorophyll-a and SPM are taken from the Schloss et al. [35].

**Fig 4:**
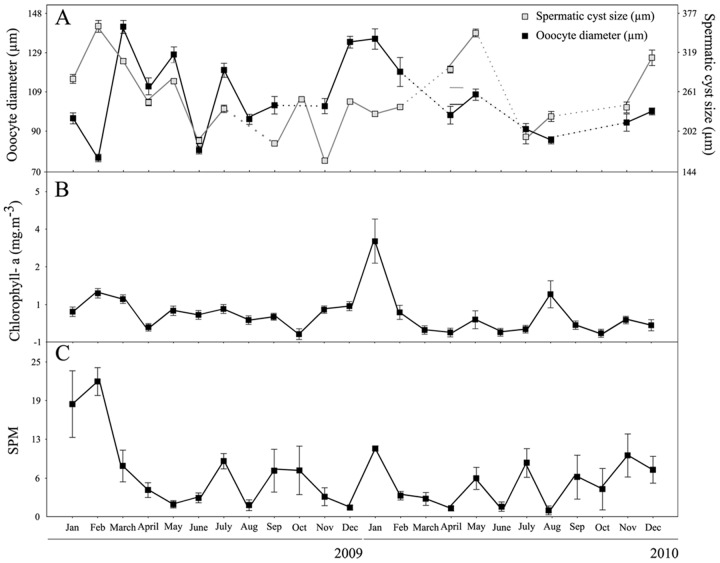
Oocytes and cysts size and environmental variation. (A) Mean oocyte feret diameter (μm) (black line) and average spermatic cyst size (μm) (dashed line). (B) Seasonal variation of Chlorophyll-a (mg.m^-3^) and (C) SPM (mg.m^-3^) data was collected by 4.7 l Niskin bottles at 5 depths (0; 5; 10; 20 and 30m) and determined by Schloss el at. (2012) Vertical bars indicate ± SE.

The pattern observed in sizes of the oocytes and sperm cysts were similar over the study months, except for February 2009 and April 2010 in which a decrease in oocyte size together with an increase in cysts were observed (Fig 4A).

### Reproductive cell location along the colonies

**Fig 5:**
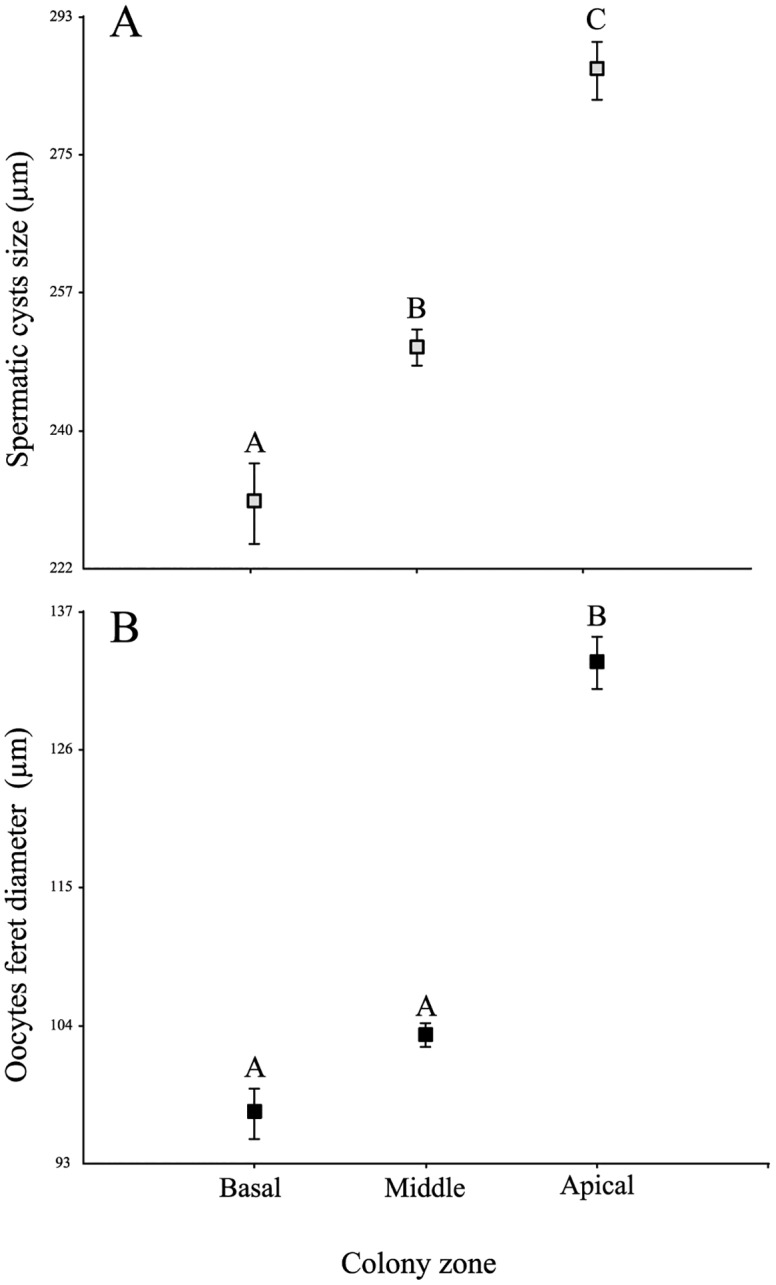
Oocytes and spermatic size distribution. (A) Mean spermatic cyst size (n = 265 basal; n = 1647 middle and n = 790 apical) and (B) oocyte feret diameter distribution in each of the colony sections: basal, middle and apical (n = 645 basal; n = 3801 middle and n = 880 apical). Vertical bars indicate ± SE. Letters indicate groups of the *a posterior* multiple range test (*p*< 0.05).

There was a significant difference in the localization of cyst sizes in the male colonies with a clear pattern of size increment from the base to the apical end: most of the small cysts were observed in the longitudinal canal at the base of the colonies, in the middle of the colonies principally medium-size spermatic cysts were present, and the largest spermatic cysts were located at the apical end (Kruskal-Wallis N = 2702 cysts, H = 80.71, *p*< 0.0001) (Fig 5). Feminine colonies showed a similar pattern oocyte diameters were significantly bigger in the apical part but showed no differences between the basal and middle parts of the colony (Kruskal-Wallis N = 5326 oocytes, H = 247.75, *p*< 0.0001) (Fig 5).

### Synchronization among feminine colonies

Oocyte's size-frequency distribution was examined in colonies from March, May and December due to the high number of female colonies examined. Colonies showed a similar distribution pattern in March 2009 (colonies 1, 4, 6 and 7), in May 2009 (colonies 1 and 2), 2010 (colonies 1, 2 and 3) and December 2009 (colonies 8 and 11) (S2 Fig). In those colonies a bimodal gamete size distribution was distinguishable, consisting of a large mode of smaller oocytes <200 μm and a smaller mode of larger oocytes (>200 μm). In several colonies (March 2009: colonies 2, 3, 5 and 9. May 2009–10: colonies 4 and 5. December 2009: colonies 3, 9 and 10 and most of the colonies in December 2010) a right-skewed distribution was exhibited,. In addition, there was a slight indication of the presence of a third modal "peak" at around 200 μm in some colonies (March 2009: colony 9. May 2009: colony 5 and May 2010: colony 6. December 2009: 2, 5 and 6) (S2 Fig).

All data underlying of this work are available in S1 File.

## Discussion

The present work provides the first year-round study of the reproductive ecology of an Antarctic pennatulid species, assessing seasonality and two-year variability in the reproductive cycle of *Malacobelemnon daytoni*.

### Seasonality and spawning

The prominent feature of oogenesis in *Malacobelemnon daytoni* was the maintenance of a large standing pool of smaller oocytes and cysts developing throughout the year, a small proportion of which matured synchronously prior to spawning. The overall results showed the presence of oocytes at the three stages (immature, stage II and mature) year-round, with a marked seasonality in number of mature oocytes, cysts and the maturity index (MSI) in both sexes, indicating a seasonal spawning. This idea(seasonal rather than continuous reproductive pattern) is reinforced by the marked lack of the oocytes >200 μm in some months. It seems oocytes get the histo-morphological maturity at 130 μm diameter but are probably ready to be released when they reach a specific size (200 μm). The observed pattern suggest that spawning could occur more than once per year, which would be a novelty for Antarctic suspension feeders reproductive strategies studied up to date. Our results suggest that the main spawning probably occurs in summer (early autumn), with a probable second one in spring in both years (September 2009 and between August-November 2010),however, with the lack of some months data, little more can be said.

Most of the Antarctic species studied to date show one reproductive peak per year, which may be more or less coupled with primary production pulses, depending on their trophic level and/or the use of reserves to fuel reproduction [3,32,37–41]. In Potter Cove the local primary production is very low to fuel the high secondary production of the benthic system which may depend on allochthonous sources [32,35,42]. Therefore in *Malacobelemnon daytoni* the reproductive seasonality should be more related to the input and content of organic matter in SPM than with primary production pulses. SPM, with different organic matter contents, can also be available for epibenthic species via sediments resuspension caused by winds (see S3 Fig) and waves, can be a food source year round or at least when the sea surface is not frozen. Measurements of SPM fluctuated during the studied months, showing peaks in July and spring 2009 (September-October), May July, September and November 2010, that can be related to resuspension events in addition to that expected in summer in both years (February 2009 and January 2010)[35]. These peaks were coincident with an increase in oocytes feret diameter and spermatic cysts size in some months (July 2009; Summer and May 2010). This relationship could be associated with an omnivorous/opportunistic diet in this species as suggested by fatty-acids profiles (Servetto et al., unpubl. data). These results suggest that *Malacobelemnon daytoni* could be also exploiting resuspended organic material (detritus and microphytobenthos) deposited at the bottom fueling reproduction decoupled from primary production pulses and probably allowing a second spawning in the year. Recent work realized by Elias-Piera et al. [43] in two Antarctic areas, the Eastern Weddell Sea and the Bransfield Strait (Antarctic Peninsula) in the southernautumn 2000, observed that a large proportion of the gorgonians diet seems to be based on sedimented and resuspended material, which supports the hypothesis that some suspension feeders deal successfully with the Antarctic winter by consuming sedimented and resuspended phytoplankton called ‘green carpets’, and microzooplankton. Another Antarctic gorgonian *Gersemia antarctica*, is able to deposit feed, this strategy involves coordinated bending of the entire colony against the substrate and was previously undescribed among soft corals [44].

There are different strategies used by Antarctic species to cope with periods of food shortage. For example, the echinoid *Sterechinus neumayeri*, the brittle star *Ophionotus victoriae* and the gorgonian *Ainigmaptilion antarticum* have extended gametogenesis of 18 to 24 mo[38,45]; in this way, the storage of material for reproduction can cover two summers when energy is diverted to gonad production, in these cases, two cohorts of oocytes can develop simultaneously. On the other hand, some benthic organisms that reproduce aseasonally or during winter can often combine strategies, such as *Odontaster validus*, which has several modes of feeding, including scavenging, suspension feeding, and active predation, also it has an oogenesis period of 18 to 24 mo and in addition can stores energy in the pyloric [46]. In the case of *Malacobelemnon daytoni*, similar to what was observed in *Cnemidocarpa verrucosa*[32], the reproductive period takes no more than 12 mo, and the storage of energy reserves to fuel reproduction has not been reported. This reproductive strategy in *Malacobelemnon daytoni* could be possible due to an opportunistic/omnivorous diet of this species, using food sources that may be quite constant via resuspension, and there for energy limitation during winter should be not so severe, at least compared with those that directly depend on the periodic pulses of primary production. And the possible presence of a second spawning in winter-early spring could be related to avoidance of larval predation, since predators are more abundant in the summer season, as is the case of the ascidian *Cnemidocarpa verrucosa* [32]. It is also interesting that these are two of the most successful species in Potter Cove [47] suggesting that reproductive strategies could be related with their ecological success.

### Inter-annual variation

There was not a strong inter-annual variability between the reproductive characteristics analyzed in 2009 and 2010, that could be related to an opportunistic feeding strategy that can make use of different food sources available via resuspension. The larger oocytes size in 2009 was the only variable that showed significant differences. This could be explained by a higher energy availability in 2009 than in 2010 and the higher concentration of SPM in 2009, although the differences were not significant. This reinforces the hypothesis that the reproductive strategy of *Malacobelemnon daytoni* is more associated with resuspension events, and with energy sources other than those provided by the local pelagic primary production. Similarly, Chiantore et al. [3] described the reproductive ecology of *Odontaster validus* from Terra Nova Bay over two successive summers, and compared the degree of inter-annual variation observed with the seastar and another opportunistic omnivore, the sea-urchin *Sterechinus neumayeri*, with the suspension feeding Antarctic scallop, *Adamussium colbecki*. There were strong inter-annual differences in the scallop, but no comparable differences in the starfish or sea-urchin. These patterns were attributed to the opportunistic feeding behavior of the two echinoderms. The absence of inter-annual variation was also observed in the reproductive cycle of the Antarctic nemertean *Parborlasia corrugatus* [40]. These observations highlight the potential importance of trophic level in reproductive strategies in polar invertebrates.

The monthly variability of oocytes size exhibited a similar pattern during the two study years (2009–2010), suggesting that the seasonal spawning could be quite constant. However, it must be considered that, at least in Potter Cove, the reproductive seasonality of *Malacobelemnon daytoni* seems to be linked to resuspension events and, in years with extensive sea-ice formation that could avoid resuspension, this association could be altered.

### Synchronization inter-sex and reproductive cell distribution

In sessile marine invertebrates with external fertilization, synchronization between males and females is a key factor in ensuring reproductive success, as the gamete dilution can play an important role in limiting the fertilization of coral eggs in the field during natural spawning [48]. The present study observed not only synchronization in the release timing of male and female gametes, but also that both sexes showed the same use of the available energy. Both reproductive cells (male and female) increased in size linked to increases in SPM, indicating a marked dependence of the energy available on the pattern of maturation of both sexes reproductive cells throughout the study period. Reproductive synchrony between males and females has been widely reported for marine invertebrates in Antarctica, including *Ophionotus victoriae*, *Odontaster validus* and *Parborlasia corrugatus* [38–40]. Spermatic cyst sizes distribution was significantly different along the entire longitudinal canal in *Malacobelemnon daytoni*. The biggest cysts size were observed in the upper part of the colony, while smaller was observed at the base of the colony. Similar that was observed with the oocytes size distribution in Servetto et al. [27], in this work we suggest that also the cysts in this species are originated in the bottom of the colony and migrate to the top of the colony for to be released by the autozooids at the apical end.

### Comparison with other octocoral species

Several authors suggest that environmental factors significantly affect gametogenesis and spawning periods in several marine invertebrates, including corals. In tropical and temperate zones, reproductive activity in octocorals has been correlated with temperature, lunar cycles and the availability of resources [49,50], while in Antarctica it has been mainly associated with pulses of primary production [51]. The reproductive strategies found in this group vary widely, from species with external fertilization, brooders, hermaphroditism, gonochorism with continuous and seasonal cycles, etc. [33,49,51,52].

Gametogenesis in *Malacobelemnon daytoni*, similar to that observed in other pennatulids [19,20], can be characterized by the maintenance of a large pool of all stages developing gametes throughout the year, a small proportion of which complete their development and are broadcast—spawned more than one time per year. The most wide spread strategy in the group includes a single spawning per year, as in *Funiculina quadrangularis* and *Pennatula phosphorea* in Scottish coastal waters, *Anthoptilum grandiflorum*in deep waters, and *Virgularia juncea*. These peaks occur in summer in *Pennatula phosphorea* and *Virgularia juncea*, or in winter in *Funiculina quadrangularis* [19,20,23,53]. The case of *Anthoptilum grandiflorum*is striking, a deep water species that reproduces just after the upper water phytoplankton bloom, as well as was observed in *Halipteris finmarchica* [15,16]. In other deep water species, such as *Kophobelemnon stelliferum*, *Pennatula aculeata* and *Anthoptilum murrayi*, a large number of oocytes at different stages were found within each colony, without a marked maturation event, suggesting no distinct seasonal reproduction [18,21,22].

The largest mature oocyte diameter in *Malacobelemnon daytoni* was approximately 350 μm feret diameter, which could be linked with a shorter gametogenesis of this species compared with other pennatulaceans, in which oocytes can reach 500 μm as in *Pennatulap hosphorea*, or 500 to 600 μm in *Ptilosarcus guerneyi*, and much larger oocytes in *Kophobelemnon stelliferum*, *Pennatula aculeata* and *Funiculina quadrangularis* (approximately 800 μm), and even more in *Anthoptilum grandiflorum* (~1.100 μm maximum diameter). In all species, the duration of oogenesis exceeds 12 months [18–20,22]. The small mature oocyte size and an oogenesis of around 12 months may help *Malacobelemnon daytoni*to reach sexual maturity at 15 mm in length, with the same fertility as a larger colony [27], while the minimum size at sexual maturity in *Pennatula phosphorea* appears to be between 55 and 90 mm (axial rod length) [20].

In conclusion, this study suggests that *Malacobelemnon daytoni* has more than one spawning event per year, which, together with the early maturity of this species [27], would bea reproductive strategy that is not common for Antarctic suspension feeders. This results are striking and may helps us to understand how this species can cope with the ice impact which is devastating for sessile benthic fauna [54]. Only two strategies could be effective to allow benthic populations to survive in highly ice impacted areas, or animals can burrow into the sediments to avoid ice removal as it could be the case of the bivalve *Laternula elliptica* or to present a high population turn-over [47]. The reproductive strategies exhibited by *Malacobelemnon daytoni* with an early sexual maturity and a probable reproductive seasonality with two peaks per year suggest that this could be the case to explain the success of this species in shallow waters affected by ice disturbances in Potter Cove.

## Supporting Information

S1 Fig Histology of colony of *Malacobelemnon daytoni* showing the different spermatogenic and oogenic classes in the longitudinal canal. (a) Oocytes in three stages (I, II and mature) (b) oocytes stage I grouped in clusters and (c) mature oocytes. (d) Spermatocyst stage I (e) stage II and (f) mature spermatocyst in the longitudinal canal. S.I: Stage I. S.II: Stage II. Ma: Mature. Nu: Nucleolus. N: Nucleo. Cl: Clusters. LC: longitudinal canal.
(TIF)

S2 Fig Oocyte size (μm) frequency distribution of individual colonies collected in (a) March 2009, (b) December and c) May 2009 and 2010. (Month, year / number of colony).
(TIF)

S3 Fig Wind speed between January 2009 and December 2010. Data were supplied by the Servicio Meteorológico Nacional (SMN) of the Argentinean Air Force at Carlini Station. datas from Schloss el at. (2012) Vertical bars indicate ± SE.
(TIF)

S1 File All data underlying this work. (XLSX)
