# Supplementary figures and images for: Reproductive Seasonality of the Antarctic Sea Pen Malacobelemnon daytoni (Octocorallia, Pennatulacea, Kophobelemnidae)
Source: PLoS One. 2016 Oct 12;11(10):e0163152. doi: 10.1371/journal.pone.0163152 (PMC5061322; doi:10.1371/journal.pone.0163152)

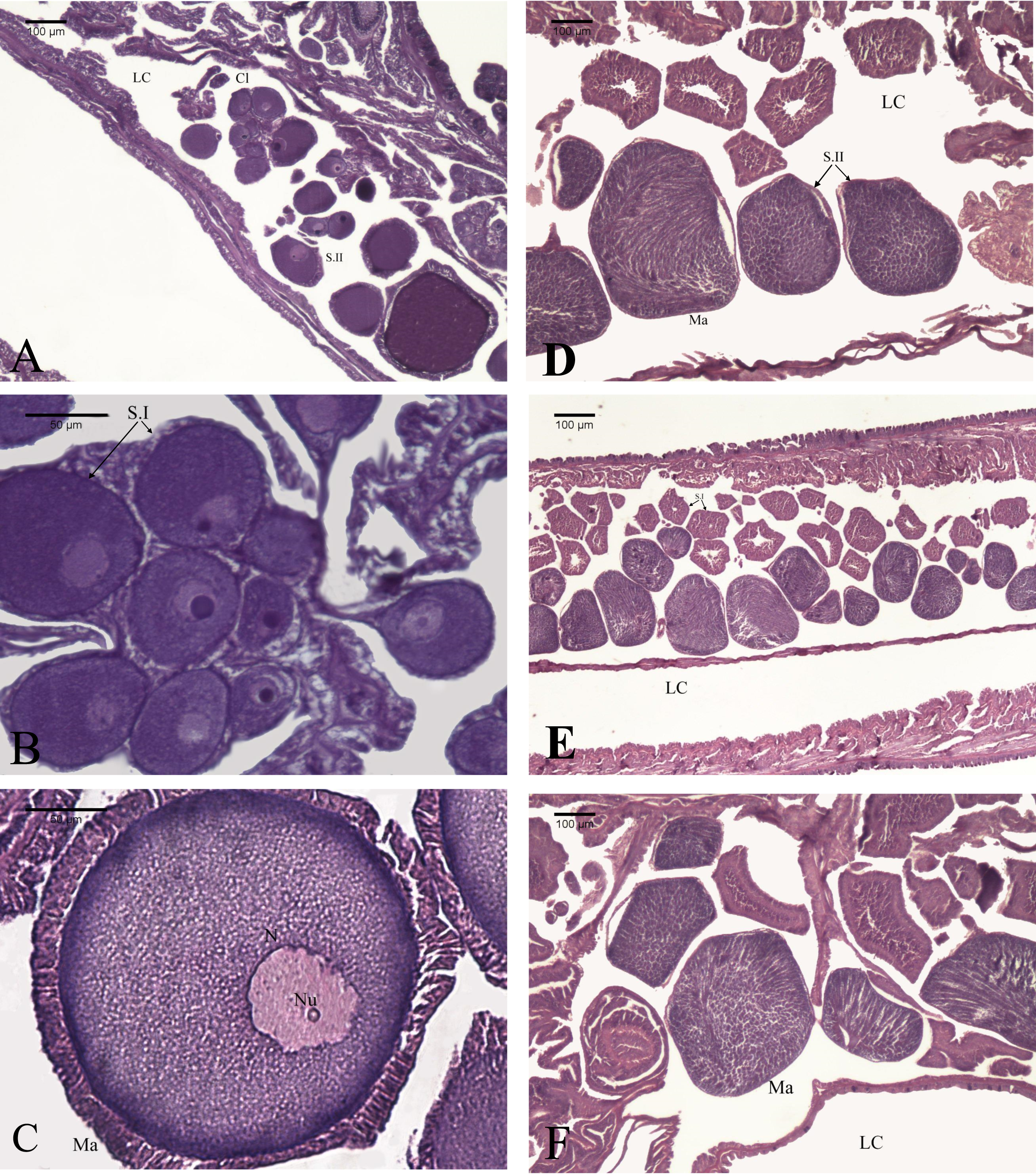

Supplement: S1 Fig — (a) Oocytes in three stages (I, II and mature) (b) oocytes stage I grouped in clusters and (c) mature oocytes. (d) Spermatocyst stage I (e) stage II and (f) mature spermatocyst in the longitudinal canal. S.I: Stage I. S.II: Stage II. Ma: Mature. Nu: Nucleolus. N: Nucleo. Cl: Clusters. LC: longitudinal canal. (TIF) [file pone.0163152.s001.tif]

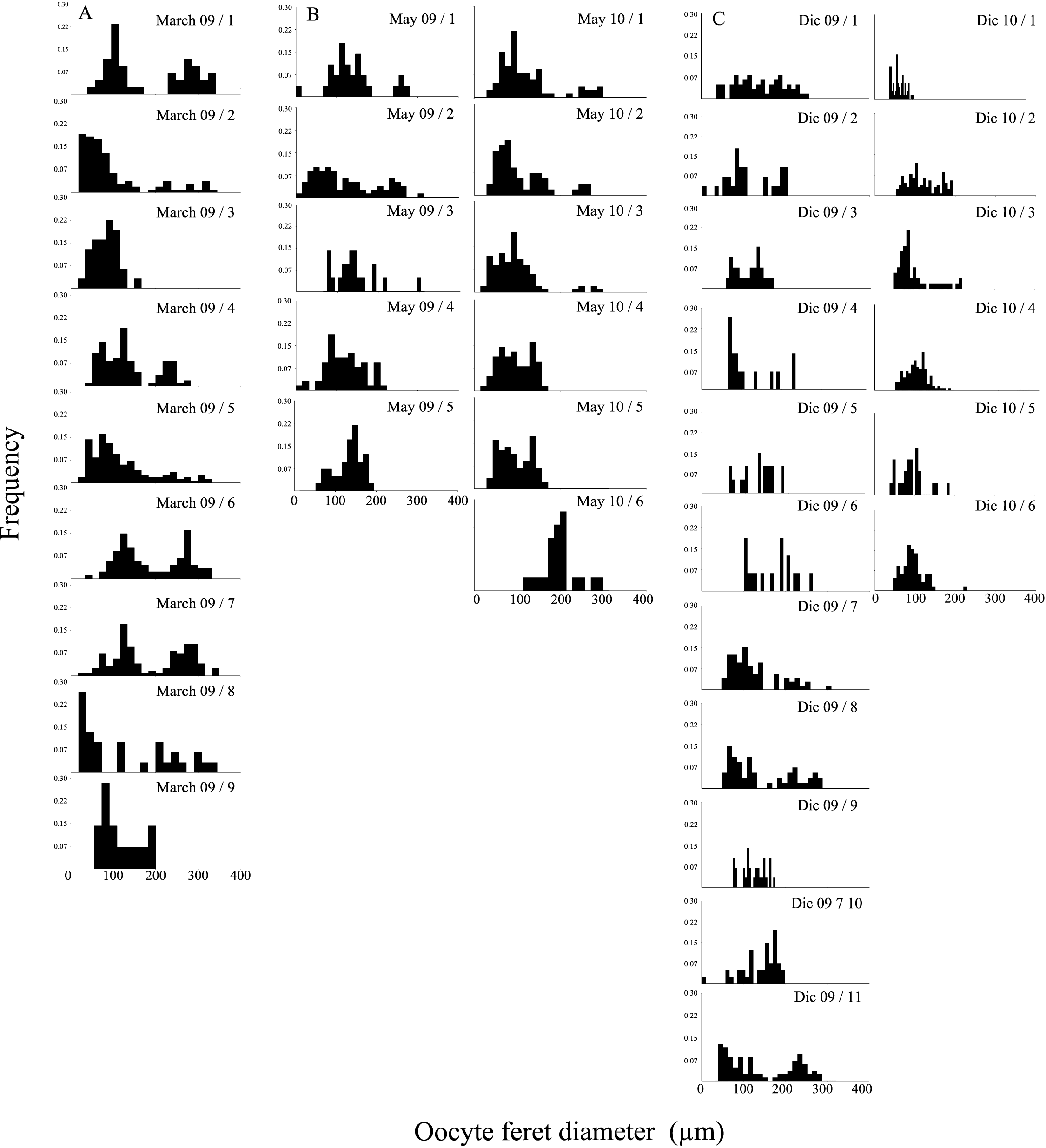

Supplement: S2 Fig — (Month, year / number of colony). (TIF) [file pone.0163152.s002.tif]

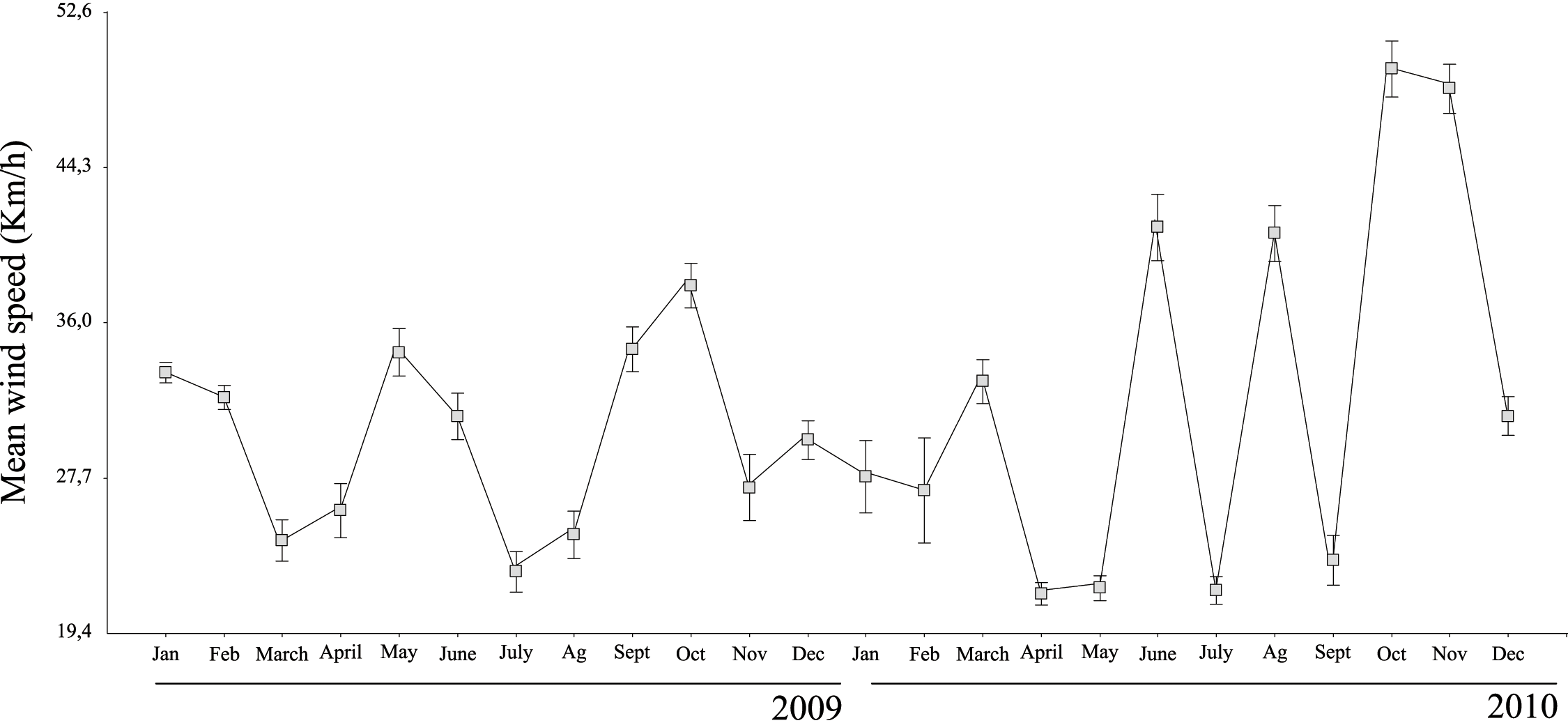

Supplement: S3 Fig — Data were supplied by the Servicio Meteorológico Nacional (SMN) of the Argentinean Air Force at Carlini Station. datas from Schloss el at. (2012) Vertical bars indicate ± SE. (TIF) [file pone.0163152.s003.tif]
